# MicroRNAs: The Missing Link between Hypertension and Periodontitis?

**DOI:** 10.3390/ijms25041992

**Published:** 2024-02-06

**Authors:** Nelia M. Rodriguez, Pía Loren, Isis Paez, Constanza Martínez, Alejandra Chaparro, Luis A. Salazar

**Affiliations:** 1 Doctoral Program in Sciences, Major in Applied Cellular and Molecular Biology, Universidad de La Frontera, Temuco 4811230, Chile; neliam.rodriguez@gmail.com (N.M.R.); isis.paez921007@gmail.com (I.P.); 2 Center for Molecular Biology & Pharmacogenetics, Department of Basic Sciences, Universidad de La Frontera, Temuco 4811230, Chile; pia.loren@ufrontera.cl; 3 Department of Oral Pathology and Conservative Dentistry, Periodontics, Faculty of Dentistry, Universidad de Los Andes, Santiago 7620001, Chile; cemartinezc@uandes.cl (C.M.); achaparro@uandes.cl (A.C.); 4 Center for Biomedical Research and Innovation (CIIB), Universidad de Los Andes, Santiago 7620001, Chile

**Keywords:** arterial hypertension, periodontitis, inflammation, miRNAs

## Abstract

Cardiovascular diseases are the leading cause of death worldwide, and arterial hypertension is a recognized cardiovascular risk factor that is responsible for high morbidity and mortality. Arterial hypertension is the result of an inflammatory process that results in the remodeling and thickening of the vascular walls, which is associated with an immunological response. Previous studies have attempted to demonstrate the relationship between oral disease, inflammation, and the development of systemic diseases. Currently, the existence of an association between periodontitis and hypertension is a controversial issue because the underlying pathophysiological processes and inflammatory mechanisms common to both diseases are unknown. This is due to the fact that periodontitis is a chronic inflammatory disease that affects the interface of teeth and surrounding tissues. However, the most likely explanation for understanding this association is related to low-grade chronic inflammation. An initial path in the study of the relationship between the mentioned pathologies is the possibility of an epigenetic influence, mediated by noncoding RNAs as microRNAs. Thus, in the present review we describe the role of microRNAs related to arterial hypertension and/or periodontitis. In addition, we identified 13 common microRNAs between periodontitis and hypertension. According to the predictions of the DIANA-mirPath program, they can regulate genes involved in 52 signaling pathways.

## 1. Introduction

Arterial hypertension is the most common chronic disease and the leading cause of heart failure, stroke, and chronic kidney disease. It has a high prevalence and is one of the main contributors to morbidity and mortality worldwide, as well as being an important risk factor for the development of cardiovascular diseases [1,2].

It must be considered that inflammation, which is a protective response to damage or infection of a cell [3], is a key feature in the initiation, progression, and clinical involvement of various cardiovascular diseases, along with the immune system. Elevated markers of inflammation, such as C-reactive protein (CRP), cytokines, and adhesion molecules, have been reported in hypertensive patients, which supports the role of inflammation in the pathogenesis of hypertension. Furthermore, in normotensive individuals, these markers have been associated with the risk of developing hypertension; in hypertensive patients, the markers have been associated with end-organ damage and a risk of future cardiovascular events. Therefore, understanding the role of inflammation in hypertension provides new insights for new therapeutic approaches that target inflammation for the treatment of hypertension and its complications [4].

Considering that the human organism functions as an integral whole, another disease commonly associated with cardiovascular disease and arterial hypertension is periodontitis, which is associated with a negative impact on the quality of life related to oral health [5]. Periodontitis is an inflammatory, infectious disease that affects the tissues that support the teeth, representing the most common cause of tooth loss in adults [6]. Furthermore, it was the 11th most prevalent global disease in 2016 [7].

It has been hypothesized that local inflammation caused by periodontitis can spread to a systemic level, influencing the subject’s inflammatory burden [8]. Recently, some authors who study the contribution of inflammation to the most common chronic diseases, such as diabetes, obesity, and cardiovascular and neurological diseases, have introduced the concept of “low-grade systemic inflammation” [9,10]. Low-grade systemic inflammation is characterized by a systemic production of inflammatory factors, constituting a risk factor for the diseases [11,12,13,14,15]. Similarly, periodontitis causes a state of low-grade systemic inflammation through the production and spillover into the bloodstream of inflammatory markers that affect endothelial function [16].

In recent years, the concept of a state of low-grade systemic inflammation as a common background of various diseases, including periodontitis, has attracted increasing scientific attention. Following this hypothesis, periodontitis could contribute, at least in part, to the development and progression of chronic systemic diseases by constantly inducing a condition of low-grade systemic inflammation, which is a silent risk factor for many of these diseases [17]. In this regard, Muñoz et al., in their study, confirmed that people with periodontitis had a 60% higher risk of suffering from hypertension than those with healthy gums. In addition, it was revealed that the markers of systemic inflammation, such as CRP and leukocyte count, were elevated in patients with periodontitis and acted as mediators of this association [18].

Even though the study of the relationship between low-grade systemic inflammation and the development of cardiovascular and periodontal diseases has increased considerably in the last decade, to date, there has been no consensus on the relevance of this association or the level of impact of one disease over the other. There are still unresolved questions, such as: What are the cellular mechanisms that promote the initiation and perpetuation of low-grade systemic inflammation? What are the molecular events involved in the clinical manifestation of systemic inflammation? Is there evidence that suggests that conditions such as high blood pressure and periodontitis have a background in low-grade systemic inflammation? Therefore, the study of the epigenetic regulation of arterial hypertension and periodontitis is interesting. The epigenetic regulation of gene expression includes DNA methylation, histone modification, and the presence of noncoding RNAs, which seem to play a prominent role in clarifying these questions. Noncoding RNAs (ncRNAs) are RNA molecules that do not encode proteins. They are functional RNA molecules that play vital roles in cellular processes, including transcription and translation. The most important ncRNAs that have been identified in regulation of gene expression in transcriptional and posttranscriptional levels include microRNAs (miRNAs), small interfering RNA (siRNA), piwi-interacting RNA (piRNA), and long ncRNA (lncRNA), among others. miRNAs are small noncoding RNAs approximately 20 nucleotides in length that regulate posttranscriptional gene expression by binding to 3′ untranslated regions (UTRs), coding sequences, or 5′ UTRs of target messenger RNA (mRNA) and leading to the inhibition of mRNA translation or degradation. miRNAs are predicted to regulate approximately 30% of the human protein-coding genome and participate in the control of the expression of genes involved in various biological processes such as apoptosis, proliferation, differentiation, and metastasis. Like other ncRNAs, miRNAs have been associated with diseases, including hypertension and periodontitis [19,20,21].

Thus, the aim of this narrative review is to provide a general description of the role of microRNAs related to arterial hypertension and/or periodontitis because understanding the principles and molecular mechanisms underlying such a complex interrelationship could lead to significant improvements in the field of customized therapeutic and diagnostic protocols.

## 2. Hypertension

Hypertension (HT) is a disease that constitutes the main risk factor for cerebrovascular disease and coronary heart disease and accounts for 54% of cerebrovascular diseases and 47% of ischemic heart disease in the world [22]. In Chile, ischemic heart disease is the leading cause of death, followed by cerebrovascular disease (42.8 and 42.7 deaths per 100,000 inhabitants, respectively) [23]. Hypertension is characterized by an increase in pressure inside the blood vessels (arteries) and is defined as systolic blood pressure figures greater than or equal to 140 mmHg and diastolic blood pressure figures less than or equal to 90 mmHg, according to the guidelines for the management of arterial hypertension of the European Society of Cardiology and the European Society of Arterial Hypertension [24]. According to the 2006 Quality of Life and Health Survey, arterial hypertension is the main chronic disease reported in the population [25,26]. The global prevalence of hypertension was estimated to be 1.13 billion in 2015. This high prevalence of hypertension is consistent throughout the world, regardless of income status; it also becomes progressively more common with advancing age. It is estimated that the number of people with hypertension will increase by 15–20% by 2025, reaching about 1.5 billion [24].

Hypertension can be classified into essential or primary HT, which corresponds to approximately 90% of cases and secondary HT in 10% of patients in whom a correctable cause can be detected. Essential HT is a polygenic disorder influenced by multiple genes or genetic combinations. On this genetic basis, a series of acquired or environmental factors exert a deleterious effect on the development of hypertension. These factors include overweight and obesity, a diet rich in salt and low in potassium, a sedentary lifestyle, alcohol consumption, and stress. The causes of secondary HT are classified as frequent and infrequent. The former includes parenchymal kidney disease, renovascular disease, primary hyperaldosteronism, sleep apnea-hypopnea syndrome, and hypertension induced by drugs, including alcohol. Rare causes include Cushing’s syndrome, hyperparathyroidism, coarctation of the aorta, and several different adrenal dysfunction syndromes [27]. The renin–angiotensin–aldosterone system, in addition to its vascular actions, induces oxidative stress at the tissue level, which specially produces endothelial dysfunction, with a breakdown of the balance between the relaxing factors of the blood vessel (nitric oxide (NO), endothelial hyperpolarizing factor (EDHF)) and vasoconstrictor factors (mainly endothelins), thus configuring the hypertensive pathology. The endothelium exerts a protective function of the arterial wall due to its anti-inflammatory, vasodilation, and antithrombotic properties. When it is dysfunctional, its vasodilator capacity decreases and there is vasoconstriction, arterial stiffness, and vascular inflammation with remodeling, which leads to an increase in total peripheral resistance and, consequently, to an increase in blood pressure [28,29].

## 3. Hypertension and Inflammation

Currently, the concept of inflammation has evolved in multiple directions to explain the different pathophysiological circumstances in which the histological and functional alteration of an organ occurs. HT is the result of an inflammatory process that includes the remodeling and thickening of the vascular walls and is associated with an immunological response. In this way, the concurrence of inflammatory cells, with forms of innate immunity and adaptive immunity [30], is described along the arterial and venous blood vessels. The immune system, inflammation, and hypertension are interrelated, as the immune system triggers an inflammatory process, in which blood pressure can rise, stimulating organ damage. Cells of the innate immune system produce reactive oxygen species (ROS), such as superoxide and hydrogen peroxide, which are intended to kill pathogens. The long-term process of inflammation increases the production of ROS, causing oxidative stress that leads to endothelial dysfunction. Effector T cells and regulatory lymphocytes, which are part of the adaptive immune system, play an important role in constricting blood vessels in hypertension [3].

It must be borne in mind that inflammation is a tissue process that is made up of a series of molecular, cellular, and vascular phenomena with a defensive purpose against physical, chemical, or biological aggressions [31,32,33]; it currently predominates in the pathophysiology of cardiovascular diseases and, among these, arterial hypertension. The inflammatory process is initiated by a gradient of chemotaxis factors expressed in the forms of intercellular (ICAM-1) and vascular (VCAM-1) adhesion molecules that result in the adhesion of monocytes to the vascular wall and their extravasation into interstitial space and the formation of a cellular assembly of macrophages, neutrophils, basophils, mast cells, and eosinophils. At the same time, the activation of a state of platelet adhesiveness and the formation of fibrin networks that configure a prothrombotic state take place on the intravascular endothelial surface. Innate immunity and adaptive immunity reactions are added to this inflammatory process. In innate immunity, complement activation, acute phase protein expression, and cytokine release take place; while adaptive immunity plays a significant role in the inflammatory response, as it helps to orchestrate the immune response and eliminate pathogens [34,35].

All of this leads to verifying that inflammation is a complex process, which occurs as a response both to infections and a variety of stimuli that cause tissue damage. If the local acute inflammatory response, which is the immediate reaction to the offending agent, is successful, the offending agent is eliminated, the damage does not spread, there are no systemic manifestations, and the tissue is satisfactorily repaired. On the contrary, if the process did not limit the damage, the initially local acute inflammation is transformed into a systemic inflammatory process that does not induce injury or loss of functionality in the infiltrated tissue, which is a distinctive feature of a low-level chronic inflammation state. In this state, the tissue shows high levels of inflammatory factors and infiltrating immune cells, while not exhibiting structural alterations or loss in its primary functions [30,36,37,38].

Recent experimental and clinical information suggests that cardiovascular disease, specifically arterial hypertension, could be the consequence of a low-grade systemic inflammatory process. This picture of systemic inflammation is characterized by an increase in the circulating levels of acute-phase proteins, such as C-reactive protein, cytokines with inflammatory activity, such as tumor necrosis factor alpha (TNF-α), and interleukins such as interleukin (IL)-1 and IL-6, which are considered markers of systemic inflammation in hypertension [34,36,39,40,41].

## 4. Inflammation and Periodontitis

Another disease that is characterized by low-grade systemic inflammation is periodontitis [42], which is the most common chronic inflammatory disease observed in humans, affecting the supporting tissues of the teeth (periodontal ligament, alveolar bone, and root cementum). In the world, its prevalence averages 33%, being more prevalent in some countries than others and affecting almost half of adults in the United Kingdom and the United States and 60% of those over 65 years of age. Recent estimates from the World Health Organization suggest that periodontitis is found in 5–20% of middle-aged adults (35–44 years) in Europe and up to 40% of older people (65–74 years). It is a major public health problem, causing tooth loss, disability, masticatory dysfunction, and poor nutritional status. Periodontitis compromises speech, reduces quality of life, and is a growing burden on the economy [43,44,45,46,47].

In Chile, there are few studies with published representative samples that assess the periodontal condition; however, studies show that there is an unfavorable periodontal condition in the population and that signs of periodontal destruction are found in adolescence, which may be one of the causes of the toothless adult population. The prevalence of this periodontal damage increases with age, in the female gender, and in the presence of smoking. In addition, it is distributed in a social gradient, with greater damage in the socially less favored groups, with important influences being family income and parents’ education [48]. Its etiology is multifactorial and it is involved in the etiopathogenesis of alcohol, insufficient diet, lack of exercise, and stress, among others [49,50].

The first step for the development of periodontal disease is the entry of bacteria, such as Gram-negative anaerobic species or their products to the periodontal tissues, causing damage to them. The bacteria produce virulence factors (e.g., lipopolysaccharide (LPS), lipoteichoic acid) that encounter the epithelial cells of the periodontal sulcus. On the other hand, the cells of the junctional epithelium (EU) produce defensins and proinflammatory cytokines, such as IL-1 and TNF, which increase the caliber of blood vessels and induce the expression of cell adhesion proteins. Subsequently, IL8, another cytokine with an important role, attracts polymorphonuclear cells (PMNs) to the site where the bacteria accumulate. These PMNs in the periodontal sulcus release reactive oxygen species and different enzymes that can cause microscopic tissue damage. Despite this, it is often possible to establish a balance of the immune response that helps resolve the inflammatory process. This immune response controls the microorganisms that accumulate in the periodontal sulcus, silently and without expressing appreciable inflammatory clinical signs. Meanwhile, the inflammatory process progresses, becoming chronic, and the degradation of the supporting tissues begins, causing the formation of the periodontal pocket, a loss of clinical attachment, and bone loss [51,52].

Among the main inflammatory mediators involved in periodontitis are IL-6 and TNF-α, although other authors also highlight the role of IL-1 and IL-8 [53,54]. Therefore, in the progression of periodontitis, the local inflammatory response is determined by the concentration of bacteria in the periodontal sulcus [55,56], the susceptibility of the host [57], unfavorable lifestyles and innate immunity mechanisms, and the release of proinflammatory molecules [58,59]. These molecules can pass into the systemic circulation and act on distant organs, accentuating an inflammatory state. Higher levels of these molecules are present in patients with periodontitis [60,61]. Likewise, periodontitis has been proposed as a factor that induces and contributes to low-grade systemic inflammation and vice versa [62,63,64]. Consequently, periodontitis may be an indirect risk factor for cardiovascular disease [65,66].

## 5. Hypertension, Periodontitis, and Inflammation

Patients with periodontitis have a higher risk of developing cardiovascular disease (CVD), as clinical markers of one pathology are associated with the other. Different studies have demonstrated better endothelial function after periodontal treatment, which is essential for vascular risk in hypertension [67,68]. Del Pinto et al. mention periodontitis as the main source of low-grade systemic inflammation. The proinflammatory signaling cascade triggered in periodontitis causes hyperpermeability of endothelial cells and the subsequent release of cytokines/interleukins (TNF-α, IL-1, IL-6) into the bloodstream, which affect endothelial function, causing alterations in the vascular structure. In addition, periodontitis has been associated with increased odds of antihypertensive treatment failure, as well as with cerebrovascular disease, coronary heart disease, and chronic kidney disease [69].

Endothelial dysfunction occurs due to the reduction in nitric oxide (NO) production caused by the release into the bloodstream of inflammatory markers such as TNF-α and IL-6. Periodontal treatment reduces systemic inflammation, allowing for the improvement of endothelial function by increasing the bioavailability of NO [16]. A meta-analysis that included 16 studies conducted in the last 15 years shows the possible influence of periodontal diseases on HT. This association can be described by common risk factors or by the diffusion of infectious and inflammatory components of periodontal lesions through the bloodstream, immune response, or glucose and lipid metabolism [70].

**Figure 1:**
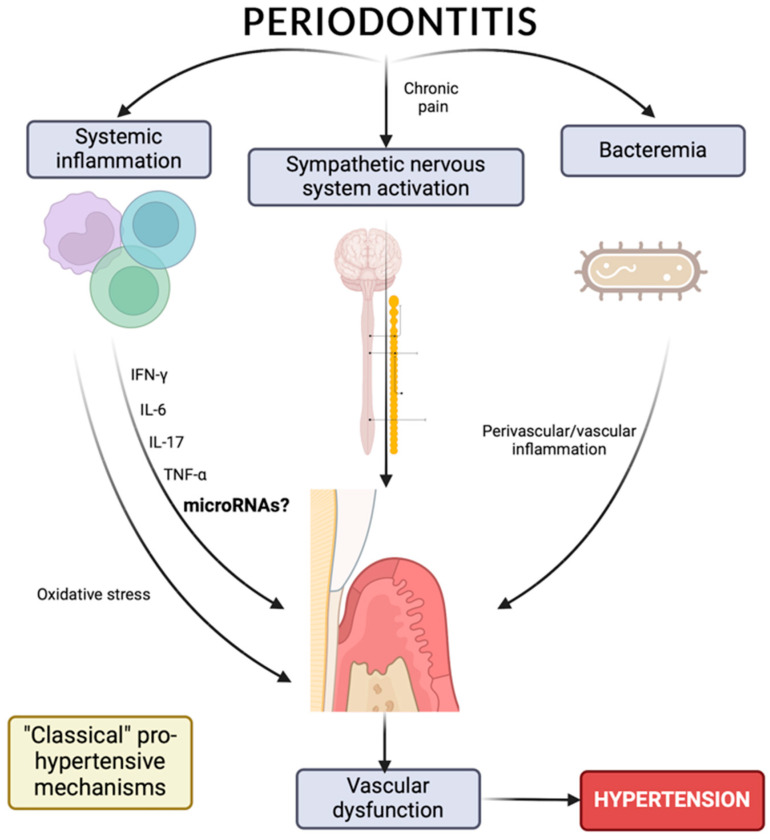
Relationship between arterial hypertension, inflammation, and periodontitis.

Although the most likely explanation for understanding the association between HT and periodontitis could be related to low-grade systemic inflammation (Figure 1), an initial path in the study of the relationship between periodontitis and HT is the possibility of a epigenetic influence, mediated by microRNAs, on the appearance and development of these pathologies; this possibility makes it interesting to study epigenetic regulation, which includes not only DNA methylation and histone modification but also the expression of miRNAs [71], which could play a prominent role in the relationship between the pathologies (Figure 1).

## 6. MicroRNAs as Risk Biomarkers for Hypertension and/or Periodontitis

miRNAs are small noncoding RNA molecules (19–25 nucleotides) that participate in epigenetic regulation at the posttranscriptional level. Genes that contain miRNAs can be located in intergenic areas, in which the regulation of their expression is produced by their own elements, or in intronic or exonic regions, in which the expression of the miRNA is closely related to the expression of the gene in question. The miRNA can act by degrading the mRNA if there is total complementarity or by repressing translation if the complementarity is partial. Because most of the target sites in the mRNA have only partial base complementarity with each miRNA, the same miRNA can interact with more than 100 different mRNAs. Furthermore, each mRNA can contain multiple binding sites for different miRNAs, giving rise to a complex regulatory network of gene expression.

miRNAs have been stably detected in different body fluids, including plasma, and are mainly transported in exosomes or microvesicles, which are associated with proteins (such as Argonaute RISC catalytic component 2 (Ago2) or nucleophosmin 1 (Npm1)), lipoproteins, and even apoptotic bodies. This suggests that circulating miRNAs would be secreted in a regulated manner in response to a stress situation, thus acting as a true intercellular communication system, regulating gene expression and the phenotype of receptor cells. In addition, they can be passively released by damaged or necrotic cells and could not be degraded by RNAses present in plasma [72].

The alteration of the expression values of miRNAs directly affects the expression of their target mRNAs; therefore, miRNAs are considered potentially causative elements of disease. Extracellular miRNAs, like their intracellular forms, are expressed in cardiomyocytes, fibroblasts, endothelial cells, and vascular smooth muscle cells; they control many of the aspects of the biology of the cardiovascular system, such as cardiac remodeling and fibrosis, apoptosis, inflammation, proliferation, angiogenesis, and metabolism. Circulating miRNAs are useful biomarkers in clinical practice due to their biological and physicochemical properties. They are highly stable, can be obtained using minimally invasive techniques, and can originate from necrotic cells or be actively secreted from living cells. The circulating miRNA profile can be highly specific depending on the tissue and the disease; it can also be altered in situations of cellular stress and pathophysiological conditions and have a long half-life within the sample [73].

Alterations in the expression of miRNAs are present in almost all cardiovascular diseases, such as in the development of ventricular hypertrophy, heart failure, and other conditions, including arterial hypertension. An example of this is miR-155, which regulates the expression of the miRNA receptor type 1 angiotensin II, which is positively related to blood pressure [74]; although other miRNAs associated with arterial hypertension have also been reported (Table 1). Similarly, in periodontal disease, miRNAs exert control over aspects of innate and adaptive immunity. As proof of this, miR-15a, miR-29b, miR-125a, miR-146a, miR-148/148a, and miR-223 were found to be upregulated as a result of periodontal disease in both human and mouse studies, while miR -92 was downregulated [75]; this constitutes examples of different miRNAs reported in the literature associated with periodontitis (Table 2). However, to date, there are no miRNA studies that link arterial hypertension with periodontitis.

## 7. Reported miRs Related to Arterial Hypertension and/or Periodontitis

Throughout our search in biological databases, such as MSDD, HMDDv3, and Arena Idb, to identify common miRNAs related to HT and periodontitis, as well as our review of the updated literature in PubMed, we were unable to find an article that reported miRNA in both pathologies at the same time. This is why they were identified as being associated with one pathology and the other independently. Thus, we found that 59 miRNAs were reported in hypertension and 79 miRNAs were reported in periodontitis (Table 1 and Table 2).

## 8. Common miRs between Hypertension and Periodontitis

**Figure 2:**
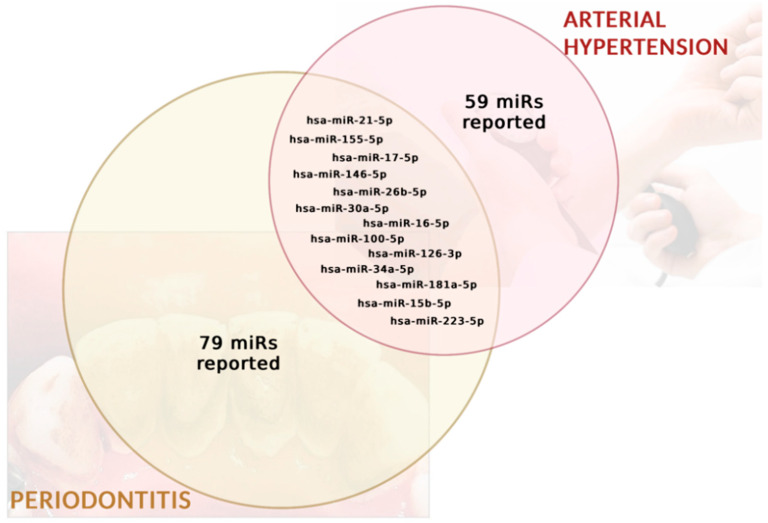
Common microRNAs between hypertension and periodontitis.

Analyzing the obtained data allowed us to find 13 miRs common to both pathologies: hsa-miR-100-5p, hsa-miR-21-5p, hsa-miR-34a-5p, hsa-miR-146a-5p, hsa-miR-26b-5p, hsa-miR-126-3p, hsa-181a-5p, hsa-miR-15b-5p, hsa-miR-223-5p, hsa-miR-16-5p, hsa-miR-30a-5p, hsa-miR-17-5p, and hsa-miR-155-5p. These miRs were reported in both arterial hypertension and periodontitis, out of a total of 59 miRs reported to be related to hypertension and 79 miRs reported to be related to periodontitis (Figure 2).

The 13 miRNAs identified as common between hypertension and periodontitis are associated with different pathophysiological processes present in both pathologies. An example of this has recently been shown, which is that miRNAs are related to blood pressure through the modulation of the immune response. Nandakumar et al. [99], in their study, identified differentially expressed miRNAs in peripheral blood to obtain information on the immune profile of hypertension and kidney disease. They found miR-17-5p, which had the most evident downregulation, miR-15b-5p, and miR-16-5p downregulated in the case group compared to controls. These miRNAs play a fundamental role in the function of immune cells. The miR-17 cluster has been found to promote T cell survival and regulate Th1 response and interleukin 10 (IL10) production in regulatory T cells. In addition, the expression of miR-15 enhances the induction of regulatory T cells; therefore, the downregulation of the miRNAs in the previous cases allows us to deduce a lower immune activation state.

The miR-17/92 group is also frequently found to be deregulated in cardiovascular, immune, and neurodegenerative diseases. The miR-17/92 cluster plays an important role in innate and acquired immunity. In addition, miR-17 participates in B cell proliferation and inhibits T cell differentiation, and high levels of this miRNA exist in different types of cancer [117]. Similarly, miR-17 inhibits transforming growth factor beta-1 proprotein-like (TGF), protein kinase, cAMP-dependent, regulatory subunit type 2 (RII), and cAMP responsive element binding protein 1 (CREB1), causing the downregulation of the Treg cell lineage, which is critical in the control of immune responses [118]. In relation to this, other authors [119] have reported in their study that other miRNAs, such as miR-15b/16, affected the in vitro induction of Treg cells.

Other important miRNAs have also been reported, such as miR-155, whose upregulation can regulate the susceptibility of human and murine CD4+ T cells to Treg cell-mediated suppression, and miR-21, which prevents CD4+ T cell apoptosis and is associated with lymphocyte oncogenesis [120]. Consistent with this, Cengiz et al. [86] found that miR-21 was significantly upregulated in the group of hypertensive patients compared to the group of normotensive subjects (*p* = 0.017). In addition, hypertensive patients had the lowest plasma levels of miR-155, which is involved in the regulation of endothelial nitric oxide synthase and suppresses angiotensin II receptor type 1. Furthermore, Vandenwijngaert et al. [96] found that miR-155 in combination with miR-425 decreased natriuretic peptide A (NPPA) expression in cardiomyocytes derived from human embryonic stem cells. Atrial natriuretic peptide (ANP) lowers blood pressure by raising the cyclic levels of 3′, 5′-guanosine monophosphate (cGMP) and inducing vasorelaxation, natriuresis, and diuresis, demonstrating that raising the ANP level is an effective treatment for atrial natriuretic peptide (ANP). Furthermore, miR-155 is considered an “Immuno-miR” for its effect on cardiovascular disease. This miRNA regulates hematopoietic cell differentiation, chemotaxis, and promotes B-cell development related to immunoglobulin production, antigen-responsive T-cell proliferation, and cytokine production [121,122].

Other functions of miR-21 are that it plays an important role in angiogenesis, apoptosis, cardiac fibrosis, and renal fibrosis [123,124]. Fernandes et al. [125] demonstrated that miR-21 levels are increased in hypertensive rats compared to normotensive rats and that physical activity reduced its expression, restoring miR-21 levels associated with revascularization in hypertension. miR-21 is the most prominent ncRNA associated with hypertension and atherosclerotic disease due to its role as “mechano-miR”, responding to arterial shear stresses. It is also one of the most dynamically regulated miRNAs in several pathophysiological processes, such as cell survival, apoptosis, and cell invasiveness [122]. Kontaraki et al. [95] revealed lower expression levels of miR-26b (6.76 ± 0.53 versus 9.36 ± 1.40, *p* = 0.037) and higher expression levels of miR-21 (2.75 ± 0.15 versus 1.82 ± 0.20, *p* = 0.002) in hypertensive patients in relation to healthy individuals.

Other miRNAs identified included miR-26, which plays a role in aspects of cardiovascular biology, such as angiogenesis, myocardial fibrosis, and atrial fibrillation, suggesting that it may have important implications in a variety of cardiovascular repair mechanisms [126,127,128]. Supporting this, Hijmans et al. [87] showed that an altered circulating expression of miR-17, miR-21, miR-34a, miR-126, and miR-146a has been linked to the pathogenesis and progression of cardiovascular disease. In their study, the circulating expression of miR-34a (9.18 ± 0.94 vs. 5.33 ± 0.91 AU) was higher, while the expression of miR-21 (1.32 ± 0.25 vs. 2.50 ± 0.29 AU), miR-126 (0.85 ± 0.10 vs. 1.74 ± 0.27 AU) and miR-146a (1.50 ± 0.20 vs. 3.10 ± 0.50 AU) were notably lower in the hypertensive versus normotensive groups, suggesting that hypertension, independent of other cardiometabolic risk factors, negatively affects the circulating profile of a subset of vessel-related miRNAs that have been associated with CVD due to their regulatory influence on inflammatory load and vascular function.

It is also recognized that miR-126 is essential in vascular homeostasis. This miRNA, when expressed in endothelial cells, decreases the expression of proteins involved in the activation and inflammation of endothelial cells, for example, high mobility group box 1 (HMGB1), adhesion molecule (VCAM)-1, C-X-C motif chemokine ligand 12 (CXCL-12) and Sprouty-related, EVH1 domain-containing protein 1 (SPRED-1). Therefore, a low expression of miR-126 promotes inflammation and endothelial cell dysfunction and reduces vascular repair capacity. In addition, it is predictive of the development of cardiovascular disease and diabetes. Therefore, in arterial hypertension, inflammation and endothelial dysfunction may be a consequence of the deregulation of miR-126 [78].

Like miR-126, miR-146a plays a central role in promoting vascular inflammation. Reduced levels of this mRNA have been associated with atherogenesis and CVD; in addition, high circulating levels of it are cardioprotective. miR-146a regulates transcription factor nuclear factor kappa B (NF-κB), which activates the transcription of proinflammatory genes, such as interluekin-6 and tumor necrosis factor α, thus increasing the inflammatory load and has been shown to be elevated in hypertension.

In contrast, an increased expression of miR-34a, another of the microRNAs, has been associated with senescence and endothelial cell dysfunction and has been linked to the development of CVD. Circulating miR-34a has been reported to be higher in adults with coronary artery disease compared with healthy controls and is, therefore, thought to favor the development of heart failure and cardiac death. Considering the characteristics of elevated blood pressure, miR-34a dysregulation may play a role in this pathology and in the deleterious cardiac effects caused by it [122].

In addition, studies conducted by Kriegel et al. [102] have aimed to identify endogenous miRNAs in endothelial cells that regulate the mRNAs encoded by genes relevant to hypertension, finding within them some of the miRNAs identified in our study. They reported a significant suppression of adrenomedullin (ADM)-encoded mRNA expression from miR-181a-5p, jagged canonical Notch ligand 1 (JAG1) from miR-21-5p, SH2B adaptor protein 3 (SH2B3) from miR-30a-5p, adrenoceptor alpha 2A (ADRA2A) from miR-30a-5p, and NADPH oxidase 4 (NOX4) from miR-100-5p. The findings indicate widespread tonic control of mRNAs encoded by genes relevant to blood pressure regulation by endothelial miRNAs. Another of the most reported miRNAs in hypertension and atherosclerotic disease is miR-223. Like miR-155, miR-223 is an immuno-miR, with the highest expression in myeloid cells, and is involved in the down-regulation of monocyte-to-macrophage differentiation [122]. Deiuliis et al. [97] found that miR-223 was elevated in the visceral adipose tissue of obese humans in the absence of hyperlipidemia and hypertension. It should also be considered that an increase in miR-223 is positively correlated with the acute incidence of ischemic stroke, although an increase in miR-223 has been shown to cause an anti-inflammatory effect in vitro [129]. In addition, several human studies show decreased miR-223 plasma levels in diabetes and CVD [130].

In addition to the important role played by microRNAs in arterial hypertension, they have been reported to play a similar role in periodontal disease, specifically in periodontitis and in the immunoinflammatory response related to it. Regarding this, a study conducted by Ogata et al. [114] reported that 17 miRNAs were overexpressed and 22 were under-expressed in inflamed gingival tissues. Among the three most overexpressed was hsa-miR-223. The upregulation of miR-223 was also observed in another investigation conducted by Stoecklin-Wasmer et al. [115]. It is also of interest to note that miR-223 is involved in many types of cancer, as well as inflammatory and autoimmune diseases [131,132].

In addition, similar investigations showed that other microRNAs, such as hsa-miR-126, were highly regulated in inflamed gingival tissues compared to healthy gingival tissues [103]. Na et al. [113] investigated the gingival tissues of individuals with periodontitis and found increased expression of miR-34a, as well as decreased expression of miR-15b. Additionally, Tang et al. [133] reported that hsa-miR-126-3p expression seemed to downregulate the inflammatory response. Interestingly, the expression of hsa-miR-126-3p was shown to be upregulated in the gingival crevicular fluid of periodontitis subjects in another investigation [134], and, as supported by other studies, expression patterns of miRNAs could be used to differentiate healthy and diseased individuals [112]. Liu et al. [106], in their study, stated that miR-17 plays an important role in osteogenic differentiation in a chronic inflammatory microenvironment, as occurs in periodontitis. On the contrary, under normal conditions, miR-17 negatively regulates osteogenic differentiation. Meanwhile, excessive levels of inflammatory cytokines decrease miR-17 levels. This miRNA downregulates lymphocyte and monocyte proliferation, as well as differentiation in the hematopoietic system, and is related to key genes that control cell cycle progression.

In periodontitis studies, the host’s immune response determines susceptibility to disease, although plaque is the main etiologic factor of periodontal disease [135]. As an example of this, Nahid et al. [136] reported increased expression of miR-146 in a mouse model of polymicrobial periodontitis. Their results suggested that miR-146, another miR identified in this study, can directly or indirectly modulate or alter the chronic periodontal pathology induced by these microorganisms because it has been shown that this miRNA plays a role in the control of the immune response. Recently, Xie et al. [112] compared the expression of miRNAs from patients with periodontal inflammation with healthy individuals, showing that in addition to miR-146, miR-155 and miR-223 were increased in inflamed gingivae. Furthermore, the lack of expression of specific miRNAs, such as miR-155, can reduce the magnitude of the immune response and lead to immunodeficiency. Finally, a constant overexpression of miR-155 or deletion of miR-146a can lead to chronic inflammation [137].

Considering that inflammation plays a key role in the fight against infection, its deregulation can cause a variety of pathologies, such as periodontitis. Therefore, it is important to regulate central elements of the adaptive and innate immune response by miRNAs that can act in antigen presentation (miR-115 and miR-181) and regulate cytokine signaling (miR-146) [138]. In this regard, it is proposed that miR-146 is an immediate early response gene that is induced by various microbial components and proinflammatory mediators [139].

O’Connell et al. [140] stated that miR-146a is a negative regulator of the immune response because during and after cellular activation, miR-146a decreases the production of inflammatory mediators such as IL-6 and TNF-α. Furthermore, it should be noted that toll-like receptors (TLRs) lead to NF-ĸB activation and recognize bacterial constituents, inducing miRNA-146a/b [79]. Unlike miR-146, miR-155 is highly induced by TLR and detects viral nucleic acids. Its expression is strongly induced by inflammatory cytokines such as IFN and TNF, concluding that this miRNA is a component of the innate immune response [141]. Similarly, the expression of several miRNAs, such as MiR-21, is elevated in activated T cells in vitro, while miR-16, miR-26, miR-30, and miR-181 were suppressed after stimulation [142]. Furthermore, based on the experiments by Chen and Lodish, it has been shown that miR-181 is related to the development of B and T lymphocytes [143]. On the other hand, Venugopal et al. [19] found that miR-21 was upregulated, whereas miR-100 was downregulated, in their study between healthy individuals and patients with chronic periodontitis. Furthermore, they discovered that NF-κB was a common target between them. It should be remembered that periodontitis is caused by the accumulation of microbial plaque, and the cell wall of these bacteria contains LPS that activates toll-like receptors (TLR). This, in turn, leads to activation of nuclear factor ĸB (NF-ĸB), which is a transcription factor that plays an important role in the inflammatory response leading to the production of proinflammatory cytokines and chemokines such as interleukin (IL)-1, IL-6, IL-8, and TNF-α.

## 9. Prediction of Target Genes and Analysis of the Signaling Pathways of the Identified miRNAs

**Figure 3:**
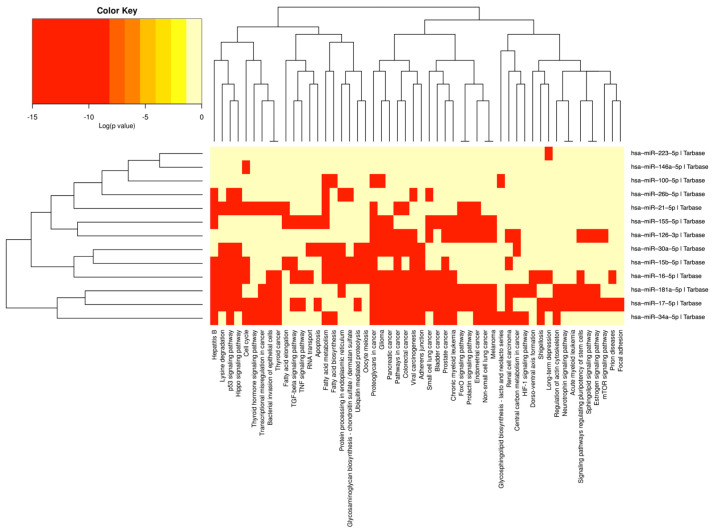
Heat map of predictive routes of signaling of miRs.

The enrichment analysis of networks and biological functions allowed us to realize that the miRs identified as common between arterial hypertension and periodontitis interact predictively with a series of genes present in different signaling pathways involved in the pathophysiology of the diseases that we are studying, which are specifically related to inflammation and with the immune response. In addition to the miRs identified by crossing the data obtained as common to arterial hypertension and periodontitis, according to the predictions of the DIANA-mirPath program, these miRs can regulate genes involved in 52 signaling pathways, as shown by the heat map in Figure 3. The identified miRs have been shown to interact predictively with genes of the Hippo signaling pathway (<1 × 10^−325^) (transforming growth factor beta receptor 1 (TGFBR1), SMAD family member 2 (SMAD2), SMAD3, SMAD4); fatty acid metabolism pathway (<1 × 10^−325^) (acyl-CoA oxidase 3, pristanoyl (ACOX3), fatty acid desaturase 1 (FADS1), FADS2); cancer pathways (2.220446 × 10^−16^) (signal transducer and activator of transcription 3 (STAT3), transforming growth factor beta receptor 1 (TGFBR1), SMAD2, NF-κB1, insulin like growth factor 1 receptor [IGF1R]), transforming growth factor-beta (TGF-β) pathway (1.051474 × 10^−9^) (TNF, TGFBR2, SMAD specific E3 ubiquitin protein ligase 2 [SMURF2]); p53 (3.815781 × 10^−11^) signaling pathway (phosphatase and tensin homolog [PTEN], insulin like growth factor binding protein 3 [IGFBP3], MDM2 proto-oncogene [MDM2]); and others.

## 10. Concluding Remarks and Future Research Directions

MicroRNAs assume an important role in the etiopathogenesis, progression, and response to treatment in arterial hypertension and periodontitis. Although no studies have identified the association between both pathologies through an epigenetic regulation mediated by miRNAs, it constitutes an interesting path of investigation to know the role that these play in the interrelationship between both diseases. The enrichment analysis of networks and biological functions demonstrates that the miRNAs identified as common between these pathologies are associated with innate and acquired immunity mechanisms, namely, inflammation and specifically, low-grade systemic inflammation, which is presented as a link between these pathologies. Understanding the biological principles and mechanisms underlying such a complex interrelationship could lead to significant improvements in the field of personalized therapeutic and diagnostic protocols. Studies should aim to determine if the association is causal or if there is a reverse causality in which hypertension leads to periodontal inflammation. Investigations should also identify the specific mechanisms involved in the relationship between periodontitis and hypertension, such as low-grade systemic inflammation, redox imbalance, neutrophil dysfunction, imbalance in T cell subtypes, oral–gut dysbiosis, hyperexpression of proinflammatory genes, and increased sympathetic outflow. Additionally, research should be conducted to explore the potential benefits of treating periodontitis in reducing systemic inflammation levels and improving cardiovascular health. Finally, studies should investigate the potential of dental offices as primary care locations for undiagnosed hypertension, as patients with moderate and severe periodontitis are more likely to be diagnosed with hypertension.

## Figures and Tables

**Table 1:** MicroRNAs related to arterial hypertension.

MicroRNAs	Model	Sample Size	Main Finding	Reference
miR-33a-5p, -144-3p	In vivo	Blood samples from 84 unrelated subjects (42 patients diagnosed with arterial hypertension and 42 normotensive subjects)	Hypertensive patients vs. control group: increased expression of miR-33a (*p* = 0.001) and miR-144 (*p* = 0.985), decreased expression of ABCA1 (*p* = 0.007) and ABCG1 (*p* = 0.550) transporters.	[76]
miR-153	In vivo	Arteries from male normotensive Wistar rats and spontaneously hypertensive rats ranging from 12 to 16 in age	Increased expression of miR 153 in the arteries of spontaneously hypertensive rats (SHR) that exhibited decreased levels of Kv7.4.	[20]
miR-145	In vivo and in vitro	Thoracic aortas from 10 26-week-old male spontaneously hypertensive rats and 10 age-matched normotensive male Wistar–Kyoto rats as control group Rat vascular endothelial cells isolated from the thoracic aortas	miR-145 functions as a key mediator in the pathogenesis of hypertension through SLC7A1 targeting.	[77]
miR-126	In vitro	HUVEC cells	Endothelial cells express miR-126, which inhibits VCAM-1 expression.	[78]
miR-146	In vitro	THP-1, U937, HL-60, WEHI-3, 293/IL-1R/MD2/TLR4, BJAB, and Mono-Mac-6 cells	The role of miR-146 in the control of toll-like receptor and cytokine signaling through the downregulation of IL-1 kinase 1 and TNF receptor-associated factor 6 protein levels.	[79]
miR-296-5p, -let-7e, hcmv-miR-UL112.	In vivo	Whole blood from 194 hypertensive patients and 97 healthy volunteers	Twenty-seven differentially expressed miRNAs. Expressions of miR-296-5p, let-7e, and a human cytomegalovirus (HCMV)-encoded miRNA, hcmv-miR-UL112 were validated in plasma samples from 24 hypertensive patients and 22 control subjects.	[80]
miR-637	In vitro	PC12 rat pheochromocytoma cells	ATP6V0A1 expression was affected by differential effects of miR-637, altering vacuolar pH and consequently CHGA processing and exocytotic secretion.	[81]
miR-1	In vivo	Hearts isolated from Wistar rats divided into six groups: control, isoproterenol, ischaemia, ischaemia–propranolol, ischaemia–propranolol-miR-1, and ischaemia–AMO (*n* = 7 for each group)	The beta-adrenergic pathway can stimulate arrhythmogenic miR-1 expression, contributing to ischemic arrhythmogenesis, and beta-blockers produce their beneficial effects in part by downregulating miR-1.	[82]
miR-483-3p	In vivo and in vitro	Hearts obtained from age and gender matched transgenic mice over-expressing the human AT1R under the mouse α-MHC promoter and corresponding littermate non-transgenic mouse lines in C3H and C57BL/6 genetic backgrounds (n = 3 for each for group) Primary human aortic smooth muscle cells	AT1R-regulated expression levels of angiotensin-1 and angiotensin-1 converting enzyme (ACE-1) proteins in VSMCs are specifically modulated by miR-483-3p.	[83]
miR-221, -222, -155	In vitro	HUVEC cells	Increased expression of miR-155 and miR-221 in HUVEC and VSMC cells. The angiotensin II type 1 receptor (AT1R) is a target of miR-155 in HUVEC. Ets-1 and its downstream genes, including VCAM1, MCP1, and FLT1, were upregulated in angiotensin II-stimulated HUVECs, and this effect was partially reversed by overexpression of miR-155 and miR-221/2	[84]
miR-208, -155	In vivo	Aortas from 8-, 16-, and 24-week-old spontaneously hypertensive rats and 8-, 16-, and 24-week-old normotensive male Wistar–Kyoto rats as control group	The miR-155 level was negatively correlated with blood pressure (r = −0.525, *p* < 0.05). The expression of miR-208 in the aorta of hypertensive rats was negatively correlated with blood pressure (r = −0.400, *p* < 0.05) and age (r = −0.684, *p* < 0.0001).	[85]
MiR-21, -122, -637, -let-7e	In vivo	Plasma samples from 30 hypertension patients, 30 white coat hypertension patients, and 30 normotensive subjects	The expression levels of MiR-21, miR-122, miR-637, and let-7e were significantly increased in the group of hypertensive subjects compared to the group of normotensive subjects (*p* = 0.017, *p* = 0.022, *p* = 0.048 and *p* = 0.013, respectively).	[86]
miR-34a, -21, -126, -146a	In vivo	Plasma samples from 15 normotensive and 15 hypertensive subjects	Circulating expression of miR-34a was higher (~170%; *p* < 0.01) whereas expression of miR-21, miR-126, and miR-146a were markedly lower (~50%, ~55%, and ~55% respectively; *p* < 0.05) in the hypertensive versus normotensive.	[87]
miR-212, -132	In vivo	Internal mammary artery with AngII receptor blockers (*n* = 16) and β-blockers (*n* = 9) from patients undergoing coronary artery by-pass graft surgery	miR-132 and miR-212 were upregulated in the heart, aortic wall, and kidney of rats with hypertension (159 ± 12 mm Hg) and cardiac hypertrophy after chronic Ang II infusion. In addition, activation of the endothelin receptor, another Gαq-coupled receptor, also increased miR-132 and miR-212.	[88]
miR-208b, -133a	In vivo	Blood and urine samples from 102 subjects with untreated newly diagnosed essential hypertension	miRNA-208b and miRNA-133a showed distinct profiles in peripheral blood cells isolated from untreated patients with newly diagnosed hypertension. Their gene expression levels revealed a strong correlation with urinary albumin excretion levels.	[89]
miR-25, -29a, -26b	In vivo	Blood samples from 104 acute Stanford type A aortic dissection + patients (of which 74 with hypertension and 30 without hypertension), and 103 age-matched acute Stanford type A aortic dissection individuals (of which 59 with hypertension and 44 without hypertension	4-miRNA (miR-25, miR-29a, and miR-155) were significantly elevated, while miR-26b was decreased in AAAD+ serum samples compared to AAAD individuals), which may serve as a non-invasive biomarker for the diagnosis of AAAD, especially for subjects with hypertension.	[90]
miR-505	In vivo and in vitro	Peripheral blood from 101 hypertensive patients and 91 healthy volunteers HUVEC cells	The plasma level of hsa-miR-505 was significantly elevated in hypertensive patients.	[91]
miR-92a	In vivo	Plasma samples from 60 healthy volunteers with normal carotid intima-media thickness (nCIMT), 60 healthy volunteers with increased CIMT (iCIMT), 60 hypertensive patients with nCIMT and 60 hypertensive patients with iCIMT	miR-92a levels showed a significant positive correlation with mean 24-h systolic blood pressure (r = 0.807, *p* < 0.001), mean 24-h diastolic blood pressure (r = 0.649, *p* < 0.001), pulse pressure 24-h mean (PP) (r = 0.697, *p* < 0.001), 24-h daytime PP (r = 0.654, *p* < 0.001), 24-h nighttime PP (r = 0.573, *p* < 0.001), CIMT (r = 0.571, *p* < 0.001) and cfPWV (r = 0.601, *p* < 0.001).	[92]
miR-29a, -29b, -29c	In vivo	Whole blood from 54 patients with untreated hypertension and 30 healthy individuals	It was observed higher expression levels of miR-29a (*p* < 0.001), miR-29b (*p* < 0.001), and miR-29c (*p* < 0.001) in hypertensive patients compared to healthy control individuals.	[93]
miR-30e, -374b, -21	-	Data obtained from Gene Expression Omnibus database	It was identified three crucial genes in the hypertensive kidney, such as COL12A1, ASPN, and SCN2A. ASPN could work in conjunction with COL12A1, and both could be targets for miR-21. SCN2A could be a new target for miR-30e and miR-374b.	[94]
miR-9, -126	In vivo	Peripheral blood mononuclear cells of patients with essential hypertension (*n* = 60) and of healthy controls (*n* = 29) for comparison	Hypertensive patients showed significantly lower miR-9 (*p* < 0.001) and miR-126 (*p* < 0.001) expression levels compared to healthy controls.	[95]
miR-425, -155	In vitro	Human embryonic stem cell-derived cardiomyocytes	The combination of miR-425 and miR-155 reduced NPPA expression to a greater extent than miR-425 or miR-155 alone, regardless of whether they separately also reduced NPPA expression.	[96]
miR-223	In vivo	Samples from lean (*n* = 19) and obese (*n* = 19) patients	miR-223 mimics the downregulation of TLR4 expression in primary macrophages while downregulating the expression of FBXW7, a well-described suppressor of Toll-like receptor 4 (TLR4) signaling.	[97]
miR-1, -133a, -26b, -208b, -499,	In vivo	Peripheral blood mononuclear cells from 152 hypertensive patients and 30 healthy volunteers	Hypertensive patients showed significantly lower miR-133a expression levels (*p* < 0.001) and higher expression levels of miR-26b (*p* = 0.037), miR-1 (*p* = 0.019), miR-208b (*p* = 0.016), miR-499 (*p* = 0.033) and miR-21 (*p* = 0.002) compared to healthy controls.	[98]
miR-17-5p, -106a-5p, -106b-3p, -15a-5p, -15b-5p, -16-5p	In vivo	Case and control pairs (N = 15 pairs) selected from individuals with hypertension treatment	MiRs from the miR-17 and miR-15 families were downregulated in progressive chronic kidney disease with high blood pressure under hypertension treatment compared with appropriate controls.	[99]
miR-361-5p	In vivo	Whole blood samples from 50 paired hypertensive patients	Significant differences in hsa-miR-361-5p and hsa-miR-362-5p expression levels between samples from patients with salt-sensitive and salt-resistant hypertension (*p* = 0.023 and 0.049, respectively)	[100]
miR-34b	In vitro and in vivo	Vascular smooth muscle cells from the medial layer of the thoracic aorta collected from a total of 36 female spontaneously hypertensive and Wistar-Kyoto rats	The negative regulatory association between miR-34b and its target, CDK6, was confirmed, which has potential as a new therapeutic target in the treatment of hypertension.	[101]
miR-181a-5p, -27, -125a-5p, -27a-3p, -21-5p, -30a-5p, -98, -92, -22-3p, -100-5p, -99b-5p	In vitro	Human dermal microvascular endothelial cells	Significant suppression of ADM-encoded mRNA expression by endogenous miR-181a-5p, ATP2B1 by miR-27 family, FURIN by miR-125a-5p, FGF5 by let-7 family, GOSR2 by miR-27a-3p, JAG1 for miR-21-5p, SH2B3 for miR-30a-5p, miR-98, miR-181a-5p, and miR-125 family, TBX3 for miR-92 family, ADRA1B for miR-22-3p, ADRA2A by miR-30a-5p and miR-30e-5p, ADRA2B by miR-30e-5p, ADRB1 by the let-7 and miR-98 family, EDNRB by the miR-92 family, and NOX4 by the miR-92 family, miR -100-5p and miR-99b-5p (*n* = 3–9; *p* < 0.05 versus scrambled anti-miR).	[102]

**Table 2:** MicroRNAs related to periodontitis.

MicroRNAs	Model	Sample Size	Main Finding	Reference
let-7a, -125b, -100, -21	In vivo	Gingival tissue samples collected from 100 individuals with healthy gingiva and 100 chronic periodontitis patients	Expression analysis revealed that let-7a and miR-21 were upregulated, whereas miR-100, miR-125b, and LIN-28 were downregulated in chronic periodontitis patients relative to healthy individuals. They found that NF-κB was a common target among all four miRNAs.	[19]
let-7a, let-7c, -130a, miR301a, miR-520d and miR-548a, miR-181b, miR-19b, miR-23a, miR-30a, miR-let7a, miR-301a	In vivo	Normal healthy gingiva and diseased gingival tissues obtained from patients undergoing periodontal treatment	miR-let-7a, let-7c, miR-130a, miR301a, miR-520d, and miR-548a were more than 8-fold up-regulated compared to healthy gingiva. MiR-181b, miR-19b, miR-23a, miR-30a, miR-let7a, and miR-301a were successfully amplified and increased significantly more in periodontitis cases than in healthy subjects.	[103]
miR-1274b, -let-7b-5p, -24-3p, -19b-3p, -720, -126-3p, -17-3p, -21-3p.	In vivo	Gingival tissue samples from 9 nonsmoker individuals with chronic periodontitis and 9 nonsmoker individuals with aggressive periodontitis	No differences were observed in the expression profiles of miRNAs between aggressive periodontitis and chronic periodontitis (*p* > 0.05). The most expressed miRNAs in both groups were hsa-miR-1274b, hsa-let-7b-5p, hsa-miR-24-3p, hsa-miR-19b-3p, hsa-miR-720, hsa-miR-126-3p, hsa-miR-17-3p, and hsa-miR-21-3p.	[104]
miR-146a, -155	In vivo	Gingival crevicular fluid from 24 healthy individuals with chronic periodontitis, 24 patients with chronic periodontitis in association with DM type 2, 24 healthy individuals with clinically healthy periodontium or 24 patients with clinically healthy periodontium in association with DM type 2	They revealed that miR-146a and miR-155 levels were significantly associated with periodontitis.	[105]
miR-17	In vivo	Healthy human tooth samples collected from 8 individuals and teeth affected by periodontal disease collected from seven periodontics clinic patients diagnosed with chronic periodontitis	They found that inflammation resulted in an inhibition of miR-17 levels, which partly reversed the differentiation potential of mesenchymal stem cells (MSCs) isolated from periodontitis-affected periodontal ligament tissue (PDLSC). They confirmed that Smurf1 is a direct target of miR-17 in PDLSC.	[106]
miR-200b	In vivo	Gingival excess tissue sample was collected from obese and normal weight subjects	The miRNA profile of gingival tissue from obese patients with periodontitis, compared to normal weight patients, showed 13 upregulated and 22 downregulated miRNAs, among which miR-200b was validated by qRT-PCR for significantly increase in obesity.	[107]
miR-21-5p, -498, -548a-5p, -495-3p, -539-5p, -34c-3p, -7a-2-3p	In vitro	LPS-treated cells from primary human periodontal ligament isolated from explanted healthy periodontal ligament	It was identified 22 upregulated miRNAs and 28 downregulated miRNAs in the LPS-treated periodontal ligament. Seven upregulated (miR-21-5p, 498, 548a-5p) and downregulated (miR-495-3p, 539-5p, 34c-3p, and 7a-2-3p) miRNAs.	[108]
miR-302a-3p	In vitro	LPS-treated cells from human mandibular osteoblast-like cells and LPS-treated cells of immortalized normal oral keratinocyte	miR-302a-3p regulates RANKL expression in HMOB within the PGE2 -IFNγ regulatory network.	[109]
miR-543	In vitro	Human periodontal ligament-derived stem cells	miR-543 was upregulated during osteogenic differentiation of human periodontal ligament-derived stem cells. Functional experiments showed that overexpression of miR-543 could enhance osteogenesis, while inhibiting miR-543 resulted in reduced formation of mineralized nodules. The ERBB2 transducer, 2 (TOB2) was identified as a target gene of miR-543.	[110]
miR-664a-3p, -501-5p, -21-3p	In vivo	Serum samples from 30 healthy patients without periodontitis and 30 patients with chronic periodontitis	The expression of hsa-miR-664a-3p, hsa-miR-501-5p, and has-miR-21-3p was higher in the periodontitis group than in the control group (*p* < 0.05).	[111]
miR-126*, -20a, -142-3p, -19a, -let-7f, -203, -17, -223, -146b, 146a, -155, -205	In vivo	Gingival tissues obtained from 10 periodontitis patients and 10 healthy subjectshasHsa-miR-126*, hsa-miR-20a, hsa-miR-142-3p, hsa-miR-19a, hsa-lehasf, hsa-has-203, has-miR-17hassa-miR-223, hsa-miR-14has hsa-miR-146a, hsa-miR-155, and hsa-miR-205 showed differential expression levels in subjects with periodontitis in relation to healthy subjects.		[112]
miR-128, -34a, -381, -15b, -211, -372, -656	In vivo and in vitro	Gingival tissues from periodontitis patients and healthy subjects THP-1 cells and CA9-22 challenged with *Porphyromonas gingivalis*	The gingival tissues of patients with periodontitis showed a higher expression of miRNA-128, miRNA-34a, and miRNA-381 and a decrease in the expression of miRNA-15b, miRNA-211, miRNA-372, and miRNA-656.	[113]
miR-150, -223, -200b, -379, -199a-5p, -214.	In vitro	Primary human gingival fibroblasts obtained from patient gingival connective tissue explants	The most overexpressed miRNAs (by >2.72 times) were hsa-miR-150, hsa-miR-223 and hsa-miR-200b, and the three most underexpressed miRNAs (by <0.39 times) were hsa-miR-379, hsa-miR-199a-5p and hsa-miR-214 in inflamed gums of patients with periodontitis.	[114]
miR-451, -223, -486-5p, -3917, -1246, -1260, -141, -1260b, -203, -210, -205	-	Data obtained from Gene Expression Omnibus database	Four miRNAs (hsa-miR-451, hsa-miR-223, hsa-miR-486-5p, hsa-miR-3917) were significantly overexpressed and 7 (hsa-miR-1246, hsa-miR-1260, hsa-miR-141, hsa-miR-1260b, hsa-miR-203, hsa-miR-210, hsa-miR-205 *) were underexpressed by >2 times in diseased gums of patients with periodontitis versus healthy gums.	[115]
miR-200c	In vivo and in vitro	12-week-old male Sprague Dawley rats microinjected with LPS-PG into the gingival sulcus Primary human gingival fibroblasts	They confirmed that local treatment with miR-200c effectively protected alveolar bone resorption in a rat model of periodontitis, by reducing the distance between the cementum-enamel junction and the alveolar bone crest and the interroot space in the second upper molar.	[116]

## Data Availability

All data are presented in this study. Data sharing is not applicable to this article.
